# Novel topical treatment for dysgeusia/ageusia: a case series report

**DOI:** 10.1007/s00520-026-11080-4

**Published:** 2026-08-12

**Authors:** Joel B. Epstein, Arezou Petrie, Geena L. Epstein, Andrei Barasch

**Affiliations:** 1 Oral Medicine Pacific, Beverly Hills, CA USA; 2 City of Hope, Duarte CA and Cedars-Sinai Medical System Los Angeles, 1500 Duarte Ave, Duarte, CA 90211 USA; 3 Jacksonville, FL USA; 4 Harvard School of Dental Medicine (ret), Boston, MA USA

**Keywords:** Dysgeusia, Ageusia, Lozenge

## Abstract

**Background:**

Management of dysgeusia has received limited study despite the significant impact taste has upon general health, nutrition, and quality of life. Taste sensation is initiated at the receptor level, leading to signal transduction and transmission to central taste centers. We assessed the potential of a topical approach to dysgeusia management.

**Methods:**

We report retrospective results of topical application of memantine, gabapentin, and monosodium glutamate (MSG) in a group of 13 patients diagnosed with dysgeusia. Compounded lozenges containing gabapentin 10 mg, memantine 2 mg, and MSG 20 mg in a base of pectin and flavoring were dissolved in the mouth three times daily. Patient-reported changes in gustation were collected. No adverse events were reported.

**Results:**

Eleven patients (85%) reported improved taste that suggests efficacy of the lozenge in improving taste function with local topical application in people that had persisting taste loss despite prior treatment trials.

**Conclusions:**

These findings support the potential of topical intervention for taste management that may have implications in preventive management and intervention for patients with dysgeusia/ageusia.

## Introduction

Gustation (taste) is a sense with complex physiology, which remains incompletely understood. Anthropologically, taste likely evolved to protect against ingestion of poisonous food and to maintain energy and nutrient intake. Current data show that a functioning taste sensation is associated with nutrition, growth and development, repair of tissue, general maintenance of homeostasis, and quality of life. Nevertheless, management of taste disorders has received relatively limited study [1–3].

Both systemic and local factors affect taste perception. Oral conditions impacting taste include quantity and quality of saliva, local microbiota, oral infection, diet, and mucosal/taste receptor function. Systemic factors associated with dysgeusia include medications, and infectious and neurologic diseases. Taste abnormalities are common in oncology due to a variety of factors including mucosal and taste bud damage induced by cytotoxic and cytoreductive therapy, targeted chemotherapy, and immune check point inhibitors [1–4].

There is a dearth of effective prophylactic and therapeutic approaches to dysgeusia. As taste receptor signaling is the initiation factor in gustation, local/topical interference with receptors and their neurologic function likely plays a critical role in this process. Hence, local topical intervention may be the logical avenue to pursue. We identified and tested three potentially efficacious chemicals for topical application, which were considered based on both their receptor interaction and potential for neuro-epithelial repair. These are:

- **Memantine** shown to reduce radiation-induced toxicities in the central nervous system (CNS) and may have a neuroprotective effect on taste [2, 5–8] by binding with open-channel N-methyl-d-aspartate (NMDA) receptors.
- **Gabapentin** acts by stabilizing sensory nerve activation and may decrease neuropathic irritation and promote recovery of normal neuronal signaling both centrally and peripherally [9].
- **Monosodium glutamate** (MSG), a receptor agonist/stimulant of T1R1/T1R3 and mGluR3/4 receptors, may enhance taste bud regeneration and neural activity in taste pathways [10]. Thus, repeated local exposure may help maintain function and prevent damage to taste and support taste recovery.

Here, we report results of a study in a cohort of 13 patients with taste loss or taste disturbance following treatment with the compounded taste lozenge.

## Methods

We report results analyzed retrospectively in a group of patients treated for ageusia/dysgeusia. Patients with diagnosed dysgeusia who had not responded to previous therapy were offered topical treatment. Demographic data and medical histories were obtained prior to initiation of lozenges. The lozenges contained gabapentin 10 mg, memantine 2 mg, and MSG 20 mg in a base of pectin and flavoring. The lozenges were dissolved in the mouth three times/day, before the main meals. The taste function was recorded using patient-reported outcomes (PROs) for five taste parameters (sour, sweet, bitter, salt, and umami). Taste PRO report was based upon the modified Scale of Subjective Total Taste Acuity (STTA), using patient visual analogue scores (VAS) for individual taste qualities and overall PRO taste report (CTCAE 4.0) [3]. Patients also reported taste fatigue, when initial taste was recognized, but followed by rapid loss of initial taste recognition with repeated intake. Assessments were performed before initiation of treatment and after 2–3 months of daily therapy, at routine follow-up visits.

## Results

Patient characteristics are shown in Table 1. Dysgeusia was related to cancer care in 9 of 13 cases, with 5 head and neck cancer patients following radiation therapy with/without chemotherapy, 4 post-hematopoietic cell transplant (HCT), 5 cases were post-COVID infection (2 with COVID alone, 2 both COVID and head and neck cancer, 1 with COVID and Sjogren disease), and 1 post-general anesthetic for upper GI surgery.

**Table 1:** Patient characteristics

Pt#	Age	Gender	Medical history	Duration of taste loss before lozenge	Cause of taste loss	Dry mouth
1	75	M	MDS, HCT 3y post	12m	GVHD	N
2	57	M	AML, HCT 9 y post	28m	GVHD	Y
3	67	F	COVID, burning mouth, *Candida*	8m	COVID, SS	Y
4	94	M	COVID	4m	COVID, Paxlovid	N
5	53	F	SCC tonsil T2N1, RT; DM II	12m	HN RT/COVID	Y
6	69	M	GERD, GA, gastritis, Barrett’s, diverticulosis	6m	Immediate onset after GA, 5% wt loss	N
7	63	M	SCC tonsil, T2Nx, (RT/CT 15 yr)	9 m post SX	HN RT/CT	Y
8	81	M	COVID, DM II, skin/oral lichen planus	30m	COVID, no taste × 2 y	Y
9	65	M	T2N1 base of the tongue, RT/CT, COVID 10m	11m	HN RT/CT improving until COVID	Y
10	56	M	SCC tonsil; T2N1; CT/RT	6m	HN RT/CT; *Candida*	Y
11	65	F	SCC oropharynx T2N2b; CT/RT	18m	HN RT/CT	Y
12	48	M	T-cell lymphoma; HCT, GVHD	6m	4 y 1 m post HCT, GVHD	N
13	75	M	CLL/MDS; HCT; GVHD	2m	GVHD	Y

*MDS*, myelodysplastic syndrome; *HCT*, hematopoietic stem cell transplant; *GVHD*, graft-versus-host disease; *AML*, acute myelogenous leukemia; *SS*, Sjogren syndrome; *SCC*, squamous cell carcinoma (head and neck cancer); stage SCC: *T*, size primary; *N*, lymph nodes; *DM II*, diabetes mellitus (type II); *GA*, general anesthetic; *GERD*, gastroesophageal reflux; *SX*, surgery; *RT*, radiation therapy; *CT*, chemotherapy; *CLL*, chronic lymphocytic leukemia

All patients were referred for dysgeusia/ageusia. Twelve patients had prior treatment for taste recovery (megestrol, 5 patients; zinc supplement 50 mg, 3 patients; sialogogue, 7 patients; CoQ10 supplement, 2 patients; Mycelex lozenge when oral candidiasis was diagnosed, 3 patients; Miracle Berry, 1 patient, and photobiomodulation treatment, 1 patient). All patients had continuing dysgeusia/ageusia despite the prior treatment trials and were then offered the taste lozenge. Taste PRO reports prior to lozenge use are shown in Table 2 and follow-up results after lozenge use are shown in Table 3. Follow-up was conducted after 2 or 3 months of lozenge use, with the last follow-up visit when PRO taste assessed, representing potential continuing effect of prior lozenge use.

**Table 2:** Patient-reported taste prior to taste lozenge use

#	Sour	Salt	Bitter	Sweet	Umami
1	1	0	0	0	0
2	2	2	2	1	0 bad
3	1	0	0	0	1 bad
4	0	0	0	0	1
5	1	1	1	1	1
6	2	2	2	1	0
7	1	0	0	2	0
8	2	1	2	2	0
9	1	1	1	1	0
10	4	4	4	3	0
11	2	1	3	2	0
12	2	2	2	1	1
13	3	3	3	3	1

Taste: 0, none; 1, 0–25% mild; 2, 25–50% Mod; 3, 50–75%; 4, 75–100%

**Table 3:** Patient-reported taste following taste lozenge use

#	Sour	Salt	bitter	Sweet	Umami	Treatment; comment	Overall change^a^ Duration of lozenge use	Last F/U after lozenge
1	3	3	2	2	3	Lozenge; significant improved all tastes	40% 6m	12m
2	4	3	4	3	3	Lozenge no change; resumed Megace 20% improved	0% 2m	4m
3	4	4	4	4	4	Taste improved 100%	100% 4 m	15m
4	4	3	4	4	4	Taste improved 80%, D/C lozenge and taste loss recurred, resumed lozenge and increase to 80% improved	80% 3m	3m
5	2	2	2	2	1	Taste fatigue, no umami	0% 3m	16m
6	4	4	4	4	4	Taste improved 80%	80% 6m	11m
7	2	3	3	4	4	Taste improved 50%	50% 3m	13m
8	4	3	4	4	3	Sweet, spicy, umami taste improved 50%, taste fatigue continued	50% 2 m	5m
9	4	4	4	4	4	Taste improved 85%; continued stable 3m	85% 3m	6m
10	4	4	4	4	4	Taste improved 90%	90% 6m	7m
11	3	3	3	3	3	Taste improved 50%	50% 4m	4m
12	4	4	4	4	4	Lozenge used 1 month stopped due to improvement and improvement continued	90% 1m	12m
13	4	4	4	4	2	Taste improved, umami reduced other tastes normal	70% 2m	2m

^a^PRO overall taste: 1 (0–25% poor), 2 (25–50% mild), 3 (50–75% moderate); 4 (75–100% good)

No adverse effects were reported. Use of the lozenge was reported convenient and well tolerated. The duration of lozenge in the mouth was 5–15 min. There was no oral sensitivity or altered taste with use of the lozenge.

Median duration of last follow-up was 9.8 months (range 2–16 months). The effect of the taste lozenge was reported by PRO: no change, 2/13(15%); < 50% improved, 4/13 (30%); 50–80% improved, 1/13 (5%); > 80% improved, 6/13 (46%); one of these patients had 100% taste recovery.

## Discussion

Dysgeusia and ageusia affect energy and calorie intake and impact a patient’s nutrition/general health and quality of life. Here, we report a case series of 13 patients treated with a compounded taste lozenge for management of taste disorders. This patient cohort consisted of a heterogenous population with dysgeusia of different etiologies, including cancer treatment and COVID infection. In these cases, a number of interventions were provided prior to the lozenge trial, with all patients presenting with persisting dysgeusia/ageusia. The results show clinically meaningful improvement in taste as recorded by PRO, with more than 80% better taste scores in 46% of patients (Tables 2 and 3). These findings support the potential for taste improvement in cases with persisting dysgeusia and continuing improved sensation reported by 12/13 (92%) of cases after stopping use of the taste lozenge.

The lozenge was initially provided for a duration of use of 2 months, and continued in some, where initial improvement was reported. One patient reported improvement after 1 month and discontinued the lozenge with continuing improvement, another patient had relapse in improvement after 2 months of lozenge use, then experienced relapse and improved after resuming the lozenge for an additional 2 months, with sustained improvement at last follow-up. The final follow-up (Table 3) represents the last patient evaluation by PRO following use of the lozenge showing continuing improvement in the majority of patients at a median follow-up of 9.8 months suggesting a durable effect of the lozenge upon taste function in most cases.

We hypothesize that the mechanism of action for the lozenge involves gabapentin binding to the α2δ subunit of voltage-gated calcium channels, release of neurotransmitters, and modulating neuronal excitability [11]. Inflammation and neuropathic changes that lead to altered signal transmission occur in taste receptors as a result of exposure to cytotoxic therapies or viral infection. By stabilizing sensory nerve firing, gabapentin may decrease neuropathic inflammation and promotes the recovery of neuronal signaling. The result appears to be an increase in flavor perception. Similarly, memantine, a NMDA receptor antagonist, modulates glutaminergic neurotransmission and is reported to reduce neuronal over-excitation and oxidative damage, which may result in neuroprotective effects, supporting regeneration and functional recovery of taste [7]. MSG is an agonist at T1R1/T1R3 and mGluR3/4 receptors and enhances taste bud signaling and regeneration, thus facilitating neural activity in taste pathways. MSG supplementation may attenuate chemotherapy-related decrease in T1R3 and mGluR3 receptors and improve taste sensation in HNC [12]. Furthermore, salivary glutamate levels have been related to pleasant taste and decreased levels are associated with increased unpleasant taste [13]. In addition, by stimulating residual taste receptors, MSG may enhance neuronal signaling and taste bud regeneration. Repeated local exposure may help maintain function by stimulating receptors and accelerating taste recovery. Additionally, local topical therapy is unlikely to have systemic side effects and therefore no potential interactions or complications are anticipated during oncological treatment.

Given the lack of a control group, these results should be considered preliminary and serve a hypothesis-generating purpose. However, these findings show the potential of topical intervention for management of dysgeusia. Further study in cancer patients with or at risk for peripheral mechanisms of dysgeusia is indicated for both prophylactic and therapeutic purposes. Topical management that affects taste receptor function may provide prophylaxis of taste loss in high-risk patients and can provide management for chronic ageusia/dysgeusia.

## Data Availability

All data supporting the findings of this study are available within the paper and its Supplementary Information.
